# A Complete Picture of the *CYP2D6* Heterogeneity in Northeastern Italian Genetic Isolates

**DOI:** 10.3390/ijms26199445

**Published:** 2025-09-26

**Authors:** Paola Tesolin, Giuseppe Giovanni Nardone, Aurora Santin, Alessandro Pecori, Romina Ruberto, Maria Pina Concas, Stefania Zampieri, Giorgia Girotto

**Affiliations:** 1 Institute for Maternal and Child Health—IRCCS, Burlo Garofolo, 34137 Trieste, Italy; paola.tesolin@burlo.trieste.it (P.T.); alessandro.pecori@burlo.trieste.it (A.P.); romina.ruberto@burlo.trieste.it (R.R.); mariapina.concas@burlo.trieste.it (M.P.C.); stefania.zampieri@burlo.trieste.it (S.Z.); giorgia.girotto@burlo.trieste.it (G.G.); 2 Department of Medicine, Surgery and Health Sciences, University of Trieste, 34149 Trieste, Italy; giuseppegiovanni.nardone@burlo.trieste.it

**Keywords:** whole genome sequencing, CYP2D6, genetic isolates, pharmacogenetics

## Abstract

The *CYP2D6* gene is a highly polymorphic pharmacogene involved in the metabolism of 25% of commonly used drugs. We aim to assess the feasibility of extracting relevant pharmacogenomic information from Whole Genome Sequencing (WGS) data and to highlight any difference in *CYP2D6* allele frequencies between the northeastern Italian and European populations. To achieve this aim, WGS was performed on two cohorts: 664 individuals from six different isolated communities (FIC) and 123 outbred Italian individuals (FOP). In silico *CYP2D6* genotyping was performed and allele frequencies from the FIC cohort were compared to those of FOP and European individuals from 1000 Genomes. Interestingly, 18 alleles identified in FIC were absent in the control cohorts. In particular, 13 individuals carried the extremely rare CYP2D6*28x2 allele, whose activity is unknown. Moreover, we identified a carrier of the CYP2D6*34x2 allele, which has never been described before. The population structure and genetic differentiation of the cohorts were investigated, revealing that the genetic isolates differ only slightly from the outbred and the European populations, but still offer new insight into *CYP2D6* heterogeneity. The findings described here will be relevant to tailoring the treatments in the northeastern Italian population.

## 1. Introduction

In recent years, Whole Genome Sequencing (WGS) has become a powerful and valuable tool commonly employed for research and diagnostic purposes, as it can detect common and rare variants, located in both exonic and intronic regions, as well as structural variants [1]. Considering WGS’s versatility and numerous possible applications, it can be hypothesized that WGS will encounter an even greater diffusion in the following years. Nevertheless, it is crucial to consider that it is still a complex analysis associated with significant time and economic efforts, which requires adequate infrastructure and experienced personnel to produce and interpret the data [1]. Considering all these factors, it is reasonable, when WGS needs to be performed, to fully exploit the information it can provide to obtain a comprehensive overview of the genetic profile of the subjects undergoing testing [2]. However, not all individuals may be open to learning about secondary findings related to their predisposition to a medical condition or disease that was previously unknown, whereas pharmacogenomic reports may be more easily accepted [3]. Indeed, pharmacogenomic data may be crucial for a patient’s health in the case of specific therapy administration and are only associated with therapy outcomes, not with disease onset or predisposition (with the exception of *RYR1* and *CACNA1S* genes [2]).

It is widely recognized that pharmacogenomics can have a positive impact on the economy, clinical outcomes, and patients’ quality of life, as its application is associated with higher therapy efficacy and lower drug adverse effects [4,5]. Several genes have been linked with therapy outcomes, and in some cases, they have strong associations supported by multiple data and studies. In this context, the *CYP2D6* gene is a critical pharmacogene involved in the metabolism of ~25% of drugs commonly employed today (e.g., paroxetine, ondansetron, codeine, imipramine, venlafaxine, etc.) [6]. It is exceptionally polymorphic: more than 177 alleles have been described, as well as several structural variants, with frequencies differing highly among populations [7,8]. Due to this high heterogeneity and significant involvement in several drug metabolisms, a careful evaluation of *CYP2D6* alleles and consequent individuals’ metabolizer status could represent a first step towards tailored therapies. To our knowledge, several studies have described the *CYP2D6* variation landscape across different populations, but none of them have evaluated *CYP2D6* heterogeneity in isolated communities. In fact, due to their intrinsic characteristics, isolated communities can aid in the identification of rare/undescribed haplotypes that can be used to (1) fully understand the value of undiscovered haplotypes and (2) provide new and insightful indications for personalized therapeutic plans.

Here, we analyzed *CYP2D6* haplotype and alleles distribution in genetic isolates leveraging WGS data of six isolated villages (Erto-Casso, Clauzetto, Resia, Sauris, San Martino del Carso (SMC), and Illegio) from Friuli Venezia Giulia (FVG), an autonomous region located in northeastern Italy at the border with Austria and Slovenia. The data obtained from this cohort were compared with those of an outbred population from the same region and those of individuals from 1000 Genomes, with a specific focus on those of European ancestry (1 KG-Eur) [9] in order to test whether the obtained findings may be applied to a broader context. With this work, we provide for the first time *CYP2D6* alleles unique to this region and evaluate potential differences in *CYP2D6* allele frequency compared to the European population, with the final aim of personalizing treatments within this particular Italian population.

## 2. Results

The *CYP2D6* genotypization from WGS was successfully carried out on 654 individuals from the six FVG isolated communities (FIC) (98%) and 120 individuals from the FVG outbred population (FOP) (98%). Moreover, 42 alleles were detected from FIC, while 18 alleles were detected in the FOP cohort (Table S1). The alleles identified in FOP are all included in the 1 KG cohort, with no significant frequency difference.

As regards the alleles detected in the FIC, 24 (57%) of those were also identified in the 1 KG cohort, while 18 alleles (43%) identified in 29 individuals were absent in the 1 KG-Eur cohort (Table 1).

In addition, 13 individuals (~2% of the cohort) carried the extremely rare CYP2D6*28x2 allele, in which two copies of the CYP2D6*28 allele are present in tandem. The allele identification has been reported in the Pharmvar database [7], but no information regarding its frequency and function is available. A kinship matrix was performed in order to determine the relationships among the 13 individuals: 3 individuals (SUB1, SUB2, SUB13) were found to be completely unrelated to the other carriers, while for the others, different degrees of kinship were present: ranging from distant relatives (~fifth degree of kinship) to parent–offspring pairs [11] (Table 2).

Regarding the other allele with multiple carriers, the two individuals carrying the CYP2D6*41x2 allele are a parent–offspring pair (kinship coefficient: 0.5062).

**Figure 1:**
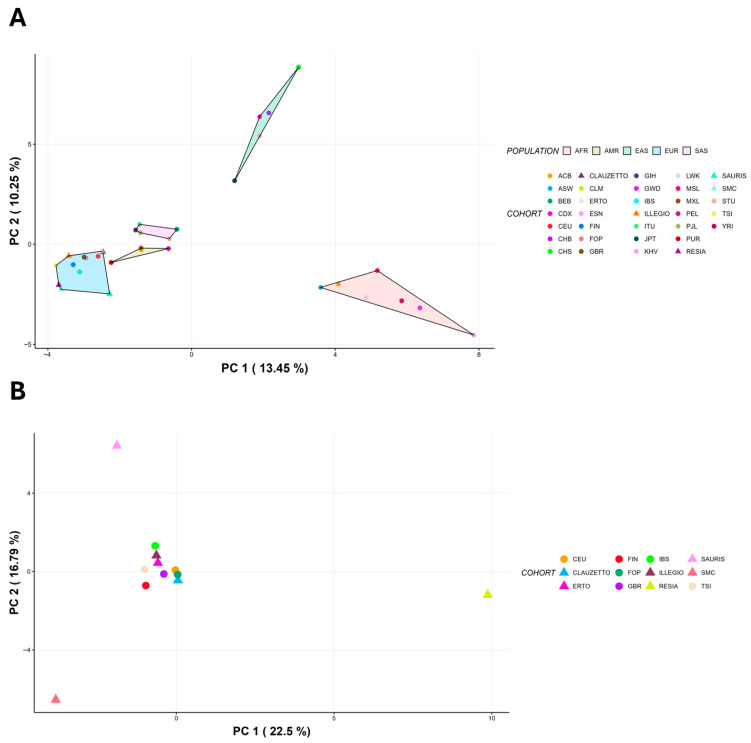
**Principal Component Analysis (PCA) based on *CYP2D6* alleles frequencies.** (**A**) PCA clustering FIC, FOP, and populations from 1 KG. FIC and FOP cluster with European populations as expected. (**B**) PCA on European populations. Resia, SMC, and Sauris cluster independently from other European populations. (ACB: African Caribbean in Barbados; ASW: African Ancestry in Southwest US; BEB: Bengali in Bangladesh; CDX: Chinese Dai in Xishuangbanna, China; CEU: Utah residents with Northern and Western European ancestry; CHB: Han Chinese in Beijing, China; CHS: Southern Han Chinese, China; CLAUZETTO: Clauzetto, FIC; CLM: Colombian in Medellin, Colombia; ERTO: Erto, FIC; ESN: Esan in Nigeria; FIN: Finnish in Finland; FOP: FVG outbred population; GBR: British in England and Scotland; GIH: Gujarati Indian in Houston, Texas; GWD: Gambian in Western Division, The Gambia; IBS: Iberian populations in Spain; ILLEGIO: Illegio, FIC; ITU: Indian Telugu in the United Kingdom; JPT: Japanese in Tokyo, Japan; KHV: Kinh in Ho Chi Minh City, Vietnam; LWK: Luhya in Webuye, Kenya; MSL: Mende in Sierra Leone; MXL: Mexican Ancestry in Los Angeles, California; PEL: Peruvian in Lima, Peru; PJL: Punjabi in Lahore, Pakistan; PUR: Puerto Rican in Puerto Rico; RESIA: Resia, FIC; SAURIS: Sauris, FIC; SMC: San Martino del Carso, FIC; STU: Sri Lankan Tamil in the United Kingdom; TSI: Toscani in Italy; YRI: Yoruba in Ibadan, Nigeria).

To test whether *CYP2D6* alleles can be used for population stratification analysis and highlight differences between FIC, FOP, and all 1000 Genomes populations, we conducted a Principal Component Analysis (PCA) on *CYP2D6* alleles frequencies. As shown in Figure 1A, FIC clusters with European populations, as expected. However, Resia, SMC, and Sauris cluster individually when performing PCA only on European populations (Figure 1B).

**Figure 2:**
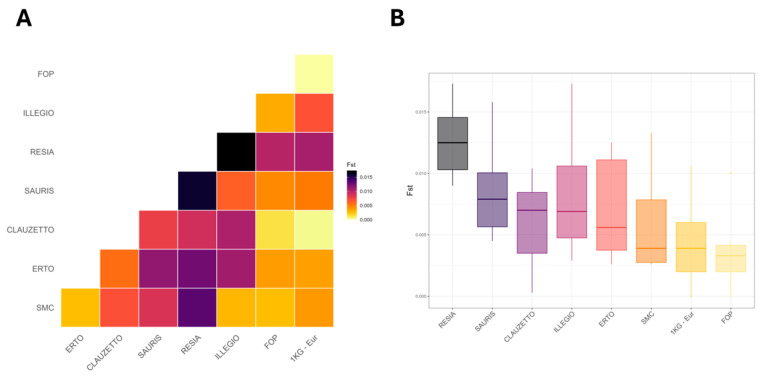
**Genetic distances for FIC, FOP and 1 KG Europeans measured with fixation index (FST).** (**A**) Heatmap of FST distances between populations. (**B**) Distribution of FST distances among tested populations.

Then, to further investigate population differentiation of FIC from European populations, an AMOVA test was performed. Despite the statistical significance (*p* = 0.001), the AMOVA test suggests a weak differentiation between FIC and European populations for *CYP2D6* alleles (ϕ_ST_ = 0.017). This is further confirmed by the low FST values between FIC, FOP and 1 KG-Eur, (Figure 2A), with Resia having the highest FST values among all tested populations (Figure 2B).

**Figure 3:**
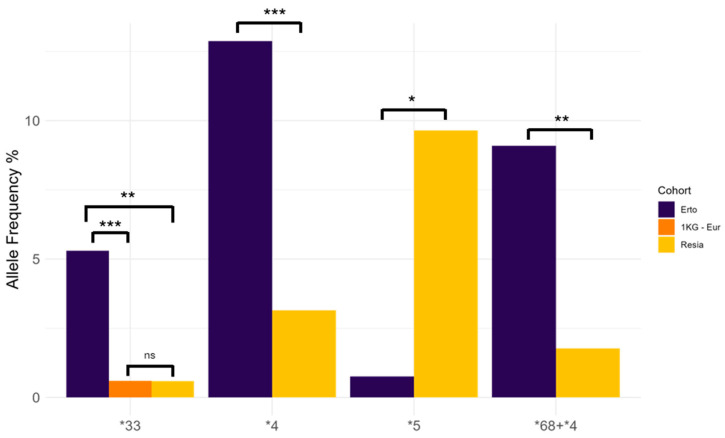
Allele frequency of significant alleles between Erto, Resia, and 1 KG Europeans. Stars specify the significance level of Bonferroni-adjusted *p*-values: ns, *p* > 0.05; *, *p* < 0.05; **, *p* < 0.005; ***, *p* < 0.0005.

To investigate differences in *CYP2D6* allele frequencies between FIC and control populations, a Kruskal–Wallis test was initially carried out, highlighting statistically significant differences among FIC, FOP, and 1 KG-Eur (*p* = 0.0005). Then, a Dunn test was conducted as a post hoc test to pinpoint significantly different groups, highlighting differences in *CYP2D6* allele frequencies between Erto and Resia (*p* = 0.04), and between Erto and 1 KG-Eur (*p* = 0.01). In particular, the differentially abundant alleles between Erto and Resia were CYP2D6*33 (*p* = 0.006), CYP2D6*4 (*p* = 0.0002), CYP2D6*68+*4 (*p* = 0.001), and CYP2D6*5 (*p* = 0.01), while only CYP2D6*33 was significantly different between Erto and 1 KG-Eur (*p* = 0.0004) (Figure 3).

**Figure 4:**
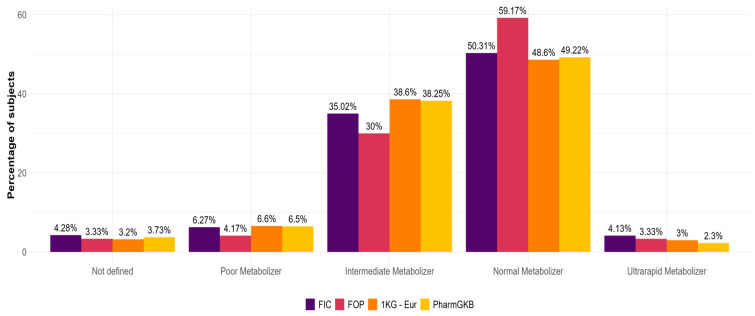
**CYP2D6 metabolizer status frequencies.** The graph shows the percentage of subjects for each class of metabolizers in FVG-isolated communities (FIC), FVG outbred population (FOP), 1 KG Europeans (1 KG-Eur) and PharmGKB Europeans (PharmGKB) [12] (percentage calculated based on the frequencies reported on the “CYP2D6 Frequency Table” reported in PharmGKB [10]).

Moreover, it was possible to define the metabolizer status for 628 individuals from FIC (95.7%), 116 subjects from FOP (96.7%), and 484 subjects from the 1 KG-Eur cohort (96.8%). The other individuals (28 from FIC, four from FOP, and 16 from the 1 KG-Eur cohort) carried alleles with unknown effects, and the metabolizer status could not be defined. Subsequently, the distribution of metabolizer classes (i.e., Ultrarapid Metabolizer -UM-, Normal Metabolizers -NM-, Intermediate Metabolizers -IM-, and Poor Metabolizers -PM-) in the different populations were compared through a proportion test using PharmGKB as reference, and no significant differences were observed (Figure 4).

In particular, as regards the 29 carriers of the 18 alleles uniquely present in our cohort, two were CYP2D6 NM, 11 presented an altered CYP2D6 metabolism (five UM, five IM and one PM), and for 16 of them, it was not possible to define the metabolizer status (alleles with unknown function).

**Figure 5:**
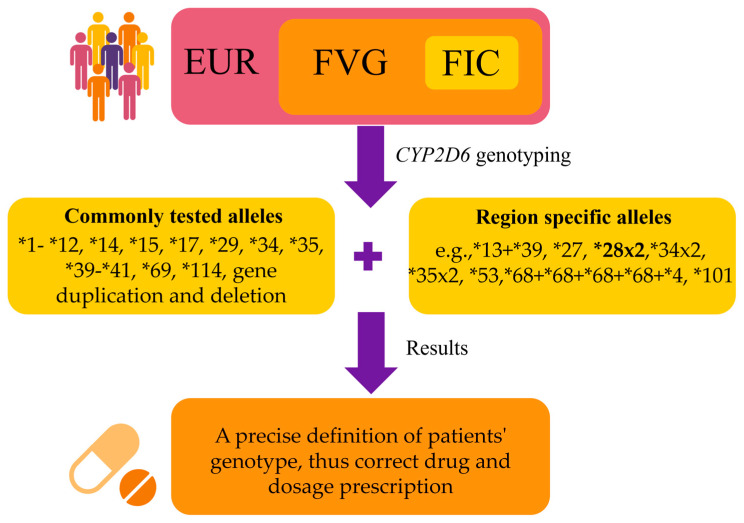
**Results summary.** The FIC population was sampled to obtain novel insight into the *CYP2D6* alleles distribution in the population from the FVG Italian region (European ancestry). By expanding the tests to include region-specific alleles in FVG (e.g., those uniquely identified in FIC with a known function, and the *28x2 allele, which appears to be more frequent in this population), it would be possible to implement personalized medicine in this population. EUR: Europeans; FVG: Friuli-Venezia Giulia Region; FIC: FVG isolated communities.

Nevertheless, the most commonly tested *CYP2D6* alleles in commercially available pharmacogenetic kits typically include alleles such as *1-*12, *14, *15, *17, *29, *34, *35, *39-*41, *69, *114, along with gene duplications and deletions [4,13]. Taking into account this consideration, the commonly performed test would have allowed us to correctly define the genotype of 84.7% (554/654) of our cohort, meaning that the information would have been lost for 15.3% (100/654) of the individuals. Thus, these findings suggest that testing region-specific alleles may allow us to solve a higher number of pharmacogenetic profiles (Figure 5).

Finally, of the 297 subjects presenting an altered *CYP2D6* metabolism (i.e., UM, IM, PM), 83 reported drug consumption. Interestingly, 8.4% (7/83 individuals) of them were using drugs with available guidelines or annotations related to the *CYP2D6* genotype. In detail, the drugs used were paroxetine [6], metoclopramide [14], nebivolol, carvedilol, timolol [15], tamsulosin, and propafenone [14].

## 3. Discussion

The *CYP2D6* gene is involved in the metabolism of commonly employed drugs, and several guidelines associate its genotype with therapy outcomes [8]. However, *CYP2D6* is a highly polymorphic gene, and its sequencing has proven to be particularly challenging [16]. Here, we performed in silico genotyping of the *CYP2D6* gene, confirming the feasibility of extracting relevant pharmacogenetic information from WGS data. As expected, the most common alleles found in Europeans appeared in our cohort (i.e., both FIC and FOP), with frequencies similar to those of the control cohort. In this context, the 1 KG-Eur cohort was employed as a standard control reference, while FOP (i.e., healthy volunteers residing in the FVG nearby regions) represent a reasonable approximation of the population living in northeastern Italy. The FIC and FOP comparison was critical considering that aggregated European data may not fully capture the genetic diversity that exists between different European countries.

No significant difference was observed between FOP and the 1 KG-Eur cohorts, possibly due to the limited sample size of FOP. However, it was interesting to identify additional rare alleles in FIC, which were not included in the 1 KG-Eur cohort, as well as the never-described CYP2D6*34x2 allele. Indeed, our findings indicate that FIC possesses sufficient genetic diversity to provide valuable insights into *CYP2D6* haplotypes, while maintaining significant similarity and serving as a bridge between the regional characteristics of the northeastern Italian population and the broader European context (i.e., FOP and the 1 KG cohorts). Consequently, clinical recommendations based on FIC data can be applied more broadly, generally extending to the whole northeastern Italian context.

Among the most relevant findings identified in FIC, it is important to highlight the CYP2D6*34 identification. Considering that the CYP2D6*34 allele is included among the normal function alleles (with a strength of evidence classified as limited) [10], it is possible to hypothesize that the CYP2D6*34x2 may be an increased function allele. Thus, considering this hypothesis and that the carrier genotype is *34x2/*39, the subject *CYP2D6* activity score would be 3 (assumed CYP2D6*34x2 activity score 2, CYP2D6*39 activity score 1). Thus, identifying the CYP2D6*34x2 allele may have important implications on the definition of the subject’s pharmacogenetic profile, considering that he should be classified as a CYP2D6 UM.

In addition, we identified several subjects carrying the CYP2D6*28x2 allele, which is extremely rare, and no information regarding its frequency or function is available. Given the relatively high percentage of carriers in FIC, we suggest that the CYP2D6*28x2 allele may be more prevalent in the northeastern Italian population. Nevertheless, it should be noted that some carriers exhibit varying degrees of kinship; therefore, expanding the FOP cohort would be essential to confirm this finding in an outbred population. CYP2D6*28x2 enrichment in FIC could depend on an ancient common ancestor shared by the carriers. Nevertheless, it is essential to highlight that the carriers belong to three different genetic isolates (i.e., SMC, Illegio, and Resia), making the hypothesis of a common ancestor less likely. Additional population studies would be required to define whether the allele originated from a common ancestor or arose independently.

Both CYP2D6*28 and CYP2D6*28x2 functionalities are unknown. However, limited findings are available regarding CYP2D6*28, which suggests that it may be a reduced-function allele (less than 50% activity compared to CYP2D6*1, measured using N-desmethyltamoxifen as a substrate) [17]. Therefore, if further studies support these findings, it can be hypothesized that CYP2D6*28x2 may be a normal function allele.

Nevertheless, these speculations should be confirmed by experimental validations. In particular, additional functional studies should be performed to characterize the activity of both CYP2D6*34 and CYP2D6*28 and those of the two duplicated alleles (i.e., CYP2D6*34x2 and CYP2D6*28x2), such as performing pharmacokinetic studies employing probe substances (e.g., dextromethorphan) [18].

The identification of rare alleles (particularly CYP2D6*28x2, considering its relatively high frequency in FIC) indicates that those may be responsible for a significant percentage of unsolved individuals’ pharmacogenetic profiles. As previously highlighted, the commonly performed test would have allowed us to correctly define the genotype of 84.7% of the FIC cohort, losing the information for 15.3% of the individuals. This suggests the need to expand the tested alleles to include not only those most commonly found in Europeans but also region-specific (i.e., northeastern Italian) additional alleles. Indeed, as shown in this study, even though the frequencies of UM, NM, IM, and PM do not differ from those reported for the reference ethnic group (Figure 4), the allelic pool in a specific cohort may vary significantly, especially for the rare alleles. Nonetheless, it can be hypothesized that the frequencies of metabolizer classes across populations may also differ once the function of rare alleles is defined. This information could be implemented into clinical practice by employing Next-Generation Sequencing tests, such as Targeted Re-Sequencing panels focusing on *CYP2D6* and eventually on other cytochromes or relevant pharmacogenes.

In conclusion, this study underscores the significant impact of WGS on pharmacogenomics and supports the potential of providing pharmacogenomic reports to patients undergoing WGS. In this case, the definition of the subject metabolizer status may have important implications for the seven individuals who are following therapeutic regimes based on CYP2D6-metabolized drugs, but also for all the 297 subjects classified as UM, IM, or PM. In the future, it will be essential to expand the screening to better define the prevalence of the haplotypes enriched in FIC in the outbred northeastern Italian population (e.g., FOP). A clear understanding of the alleles most commonly found in northeastern Italians will have important implications for optimizing treatment strategies in this specific population.

## 4. Materials and Methods

### 4.1. Cohort Selection and Genetic Analysis

The FIC cohort was composed of 664 subjects from six different northeastern Italian genetic isolates from the FVG genetic park: Clauzetto-77; Erto-67; Illegio-80; Resia-257; SMC-104; and Sauris-79 [19]. The age of the participants ranged from 4 to 86 years old. All participants were asked to indicate whether they were currently using any drugs and if so, to provide a list of the substances used. The FOP cohort included 123 additional subjects from the FVG region, included as an outbred control population. A blood sample was collected from all participants, and the extracted DNA was used to prepare the library for WGS with the Illumina DNA Prep Kit (Illumina Inc., San Diego, CA, USA), according to the manufacturer’s protocol. Subsequently, WGS was carried out using the Illumina NovaSeq 6000 platform, which employed the sequencing by synthesis method. After sequencing, BAM files were generated through the following steps: (1) Fastq trimming and quality control was performed using, respectively, Fastp [20] and Fastqc software (version 0.11.9) [21]; and (2) alignment to the Human Genome Reference build 38p.13 (GRCh38p.13) was performed using the BWA software (version 2.1) [22]. Subsequently, refinement of the BAM files was conducted through PCR duplicate removal using Sambamba (version 1.0) [23] and base quality score recalibration was performed using GATK (version 4.1.9.0) [24].

*CYP2D6* in silico genotyping from previously generated BAM files was performed using Cyrius [9]. *CYP2D6* haplotypes and allele frequencies from FIC were compared to those of 1 KG-Eur [9] and to the FOP cohort previously genotyped using Cyrius.

A kinship matrix was computed to evaluate the relatedness relationships among the individuals analyzed, based on identity-by-descent estimates (i.e., employing the -genome command), using the PLINK v1.90 software [25].

For this work, all the statistical analyses were carried out using the R statistical software R 4.4.2 (https://www.r-project.org/), and statistical significance was defined at *p* < 0.05. To investigate population structure, PCA on *CYP2D6* allele frequencies was carried out using the “*prcomp()*” R function. Furthermore, AMOVA tests [26] and the fixation index (FST) [27] and the “*genet.dist()*” R function from the hierfstat package [28] were used to investigate genetic differentiation. To highlight differences in *CYP2D6* allele frequency between cohorts, the Shapiro test, Kruskal–Wallis test, and Dunn test were used to, respectively, test for data normality, check for differences in allele frequency between cohorts, and employ a pairwise multiple-comparison between cohorts. Bonferroni correction was used to correct for multiple testing. Finally, a proportion test was used to investigate differentially present *CYP2D6* alleles among significantly different cohorts and compare percentages of individuals within metabolizer classes among cohorts, setting the percentages available in PharmGKB as reference.

### 4.2. Activity Score and Metabolizer Status

The Activity Score system [29] was employed to classify individuals between UM, NM, IM, and PM. In detail, the respective activity score was assigned to each allele, as defined by the “CYP2D6 Allele Functionality Table” present in the PharmGKB database [10]. Subsequently, for all the subjects, the activity scores of the two alleles were added together to define a final activity score, which was used to classify the individuals as follows:

- Not defined: carrying at least one allele with an unknown function;
- UM: activity score equal to or above 2.5;
- NM: activity score between 1.25 and 2.25 inclusive;
- IM: activity score between 0.25 and 1 inclusive;
- PM: activity score equal to 0.

## Figures and Tables

**Table 1:** **Rare alleles present in FIC and absent in the 1 KG-Eur cohort.** For each allele, the associated function and the allele count in the FIC cohort are reported. All the alleles were identified in the heterozygous state. To define the frequency in different populations, we employed the “Frequency Table” reported in the PharmGKB database [10]. The population in which the allele is most frequent and its corresponding frequency are reported. (AC: allele count; AF: allele frequency; NA: not available).

Allele	Function	AC in FIC	Population with the Highest AF (PHAF)	AF in the PHAF
*1x3	Increased	1	Sub-Saharan African	0.002
*1x4	Increased	1	Sub-Saharan African	0.002
*4.013	No function	1	NA	NA
*6x2	No function	1	Latino	0.00011035591
*13+*39	Normal	1	NA	NA
*27	Normal	1	Sub-Saharan African	0.0033542388
*28x2	Unknown	13	NA	NA
*34	Normal	1	European	0.010744718
*34x2	Unknown	1	NA	NA
*35x2	Increased	1	Latino	0.00093648373
*39	Normal	1	Near Eastern	0.026844444
*41x2	Decreased	2	Near Eastern	0.004381261
*53	Normal	1	Latino	0.005
*68+*68+*68+*68+*4	No function	1	NA	NA
*74	Unknown	1	Sub-Saharan African	0.0013710798
*101	No function	1	Central/South Asian	0.003
*116	Unknown	1	NA	NA
*121	Unknown	1	African American/Afro-Caribbean	0.003

The alleles identified in FIC reported in Table 1 can all be considered rare worldwide, but their frequency appears to be higher in populations other than the European populations (with the exception of CYP2D6*34). Notably, one individual carried the CYP2D6*34x2 allele (i.e., a duplication of the CYP2D6*34), which has never been described before and is not included in public repositories [7,10].

**Table 2:** Kinship matrix for the 13 carriers of the CYP2D6*28x2 allele. A kinship coefficient of zero indicates that the two subjects are unrelated, while the symbol “-” is reported when the two compared individuals are the same person. Values different from 0 are reported in bold. For all the subjects, the FIC village of origin is reported. (SMC: San Martino del Carso).

		SMC	Illegio	Illegio	Illegio	Illegio	Illegio	Illegio	Resia	Resia	Resia	Resia	Resia	Resia
		**SUB1**	**SUB2**	**SUB3**	**SUB4**	**SUB5**	**SUB6**	**SUB7**	**SUB8**	**SUB9**	**SUB10**	**SUB11**	**SUB12**	**SUB13**
SMC	**SUB1**	-												
Illegio	**SUB2**	0.0000	-											
Illegio	**SUB3**	0.0000	0.0000	-										
Illegio	**SUB4**	0.0000	0.0000	**0.0406**	-									
Illegio	**SUB5**	0.0000	0.0000	**0.0579**	**0.4850**	-								
Illegio	**SUB6**	0.0000	0.0000	**0.0245**	**0.0227**	**0.0437**	-							
Illegio	**SUB7**	0.0000	0.0000	**0.0238**	**0.1435**	**0.2375**	**0.0398**	-						
Resia	**SUB8**	0.0000	0.0000	0.0000	0.0000	0.0000	0.0000	0.0000	-					
Resia	**SUB9**	0.0000	0.0000	0.0000	0.0000	0.0000	0.0000	0.0000	**0.0411**	-				
Resia	**SUB10**	0.0000	0.0000	0.0000	0.0000	0.0000	0.0000	0.0000	**0.0507**	0.0000	-			
Resia	**SUB11**	0.0000	0.0000	0.0000	0.0000	0.0000	0.0000	0.0000	**0.4710**	**0.0407**	**0.0310**	-		
Resia	**SUB12**	0.0000	0.0000	0.0000	0.0000	0.0000	0.0000	0.0000	**0.0619**	**0.0391**	**0.4853**	0.0000	-	
Resia	**SUB13**	0.0000	0.0000	0.0000	0.0000	0.0000	0.0000	0.0000	0.0000	0.0000	0.0000	0.0000	0.0000	-

## Data Availability

The data underlying this article will be shared upon reasonable request to the corresponding author.

## Supplementary Materials

The following supporting information can be downloaded at https://www.mdpi.com/article/10.3390/ijms26199445/s1.
